# Measurement approaches in continuum of care for maternal health: a critical interpretive synthesis of evidence from LMICs and its implications for the South African context

**DOI:** 10.1186/s12913-018-3278-4

**Published:** 2018-07-11

**Authors:** Mamothena Carol Mothupi, Lucia Knight, Hanani Tabana

**Affiliations:** University of the Western Cape, Robert Sobukwe Road, Bellville, 7535 South Africa

**Keywords:** Continuum of care, Maternal health, Critical interpretive synthesis, Maternal health services, Low and middle income countries, South Africa

## Abstract

**Background:**

Global strategies recommend a continuum of care for maternal health to improve outcomes and access to care in low and middle income countries (LMICs). South Africa has already set priority interventions along the continuum of care for maternal health, and mandated their implementation at the district health level. However, the approach for monitoring access to this continuum of care has not yet been defined. This review assessed measurement approaches in continuum of care for maternal health among LMICs and their implications for the South African context.

**Methods:**

We conducted a critical interpretive synthesis of quantitative and qualitative research sourced from Academic Search Complete (EBSCO), MEDLINE (Pubmed), Cambridge Journals Online, Credo Reference and Science Direct. We selected 20 out of 118 articles into the analysis, following a rigorous quality appraisal and relevance assessment. The outcomes of the synthesis were new constructs for the measurement of continuum of care for maternal health, derived from the existing knowledge gaps.

**Results:**

We learned that coverage was the main approach for measuring and monitoring the continuum of care for maternal health in LMICs. The measure of *effective coverage* was also used to integrate quality into coverage of care. Like coverage, there was no uniform definition of effective coverage, and we observed gaps in the measurement of multiple dimensions of quality. From the evidence, we derived a new construct called *adequacy* that incorporated timeliness of care, coverage, and the complex nature of quality. We described the implications of adequacy to the measurement of the continuum of care for maternal health in South Africa.

**Conclusions:**

Critical interpretive synthesis allowed new understandings of measurement of the continuum of care for maternal health in South Africa. The new construct of *adequacy* can be the basis of a new measure of access to the continuum of care for maternal health. Although adequacy conceptualizes a more holistic approach, more research is needed to derive its indicators and metrics using South African data sources.

**Electronic supplementary material:**

The online version of this article (10.1186/s12913-018-3278-4) contains supplementary material, which is available to authorized users.

## Background

One of the health and well-being targets of the Sustainable Development Goals is to reduce the global maternal mortality ratio to less than 70 deaths per 100,000 live births [1]. Health service delivery strategies such as the continuum of care are touted as important components of strong health systems, needed to prevent and reduce maternal morbidity and mortality [2–4]. The continuum of care ensures that services are provided in an integrated manner to reduce duplication of effort and save costs [3]. It is defined as,

*“…access to care provided by families and communities, by outpatient and outreach services, and by clinical services throughout the lifecycle, including adolescence, pregnancy, childbirth, the postnatal period, and childhood. Saving lives depends on high coverage and quality of integrated service-delivery packages throughout the continuum, with functional linkages between levels of care in the health system and between service-delivery packages, so that the care provided at each time and place contributes to the effectiveness of all the linked packages.”* ([3], p1359)

**Fig. 1:**
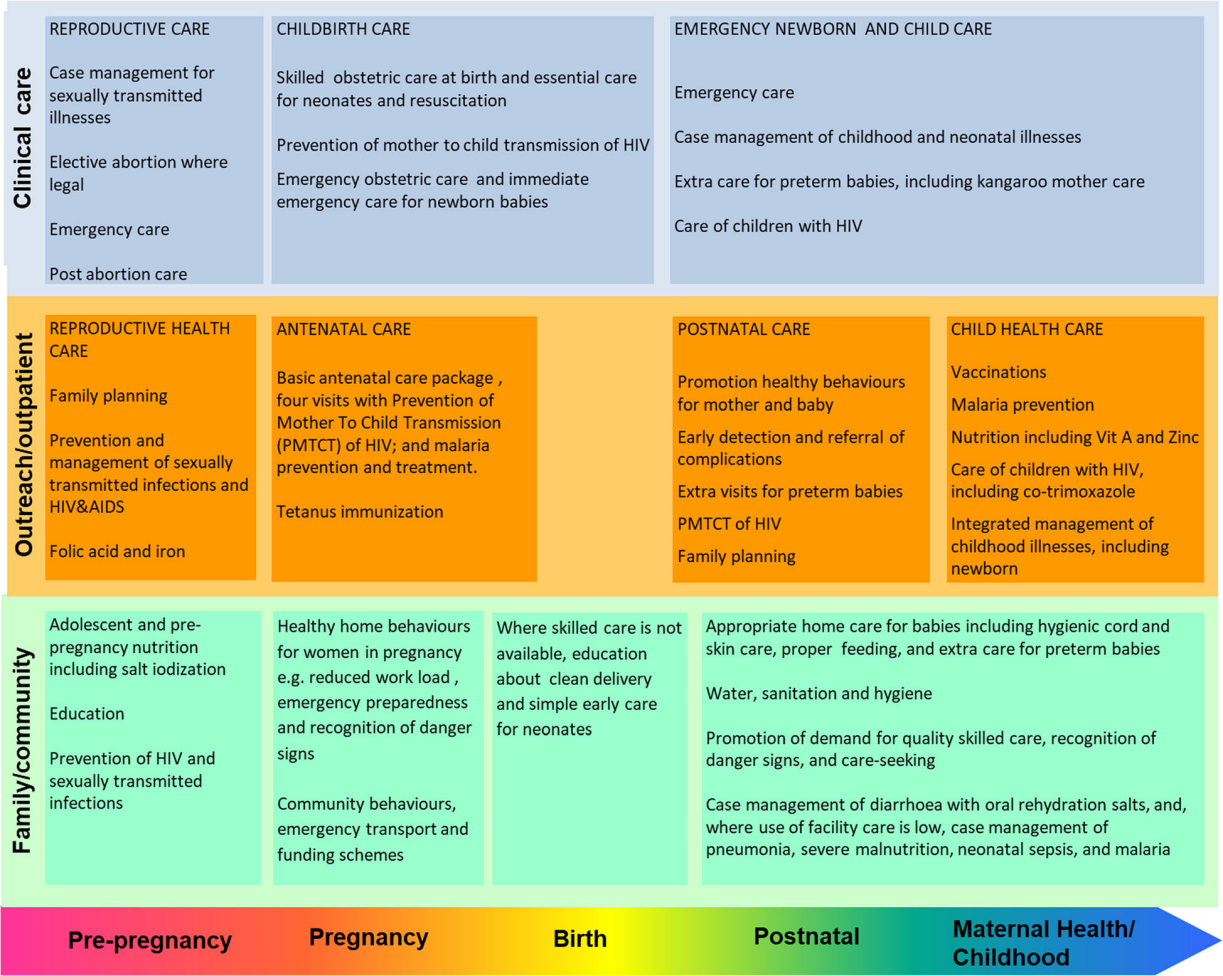
Continuum of care for maternal and child health in low and middle income countries (Adapted from [3])

The continuum of care framework for maternal and child health in low and middle income countries is illustrated in Fig. 1. This framework illustrates the health service interventions to be provided throughout the lifecycle, as well as the social dimensions of care.

**Fig. 2:**
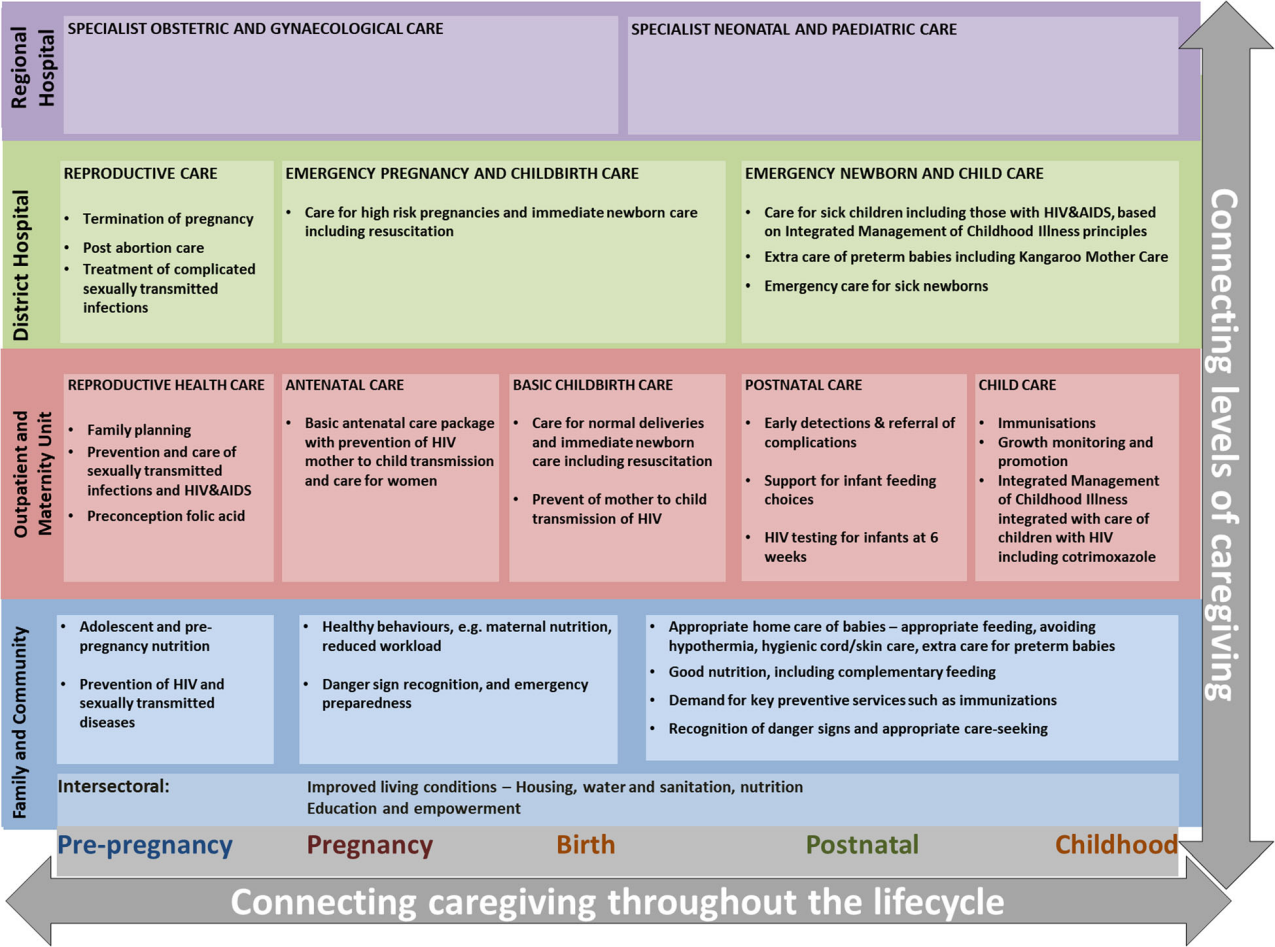
The continuum of care for maternal and child health framework as adapted to the South African health system [7]

In South Africa, the continuum of care is expected to lower the maternal mortality ratio from the current estimated 140 deaths per 100,000 live births in 2013 [5], to less than 100 deaths per 100,000 live births by 2019 [6]. The Department of Health in South Africa has outlined its own maternal (and child health) continuum of care framework which is similar to the Kerber framework (Fig. 2) [7]. The framework adds care at the specialist/regional hospital levels, and isolates social determinants of health as “intersectoral factors”. In addition, the National Strategic Plan for Maternal, New-born, Child and Women’s Health and Nutrition 2012–2016 of the department has set out priority interventions for up to six days post-delivery [8]. However, there were still gaps in defining pre-pregnancy, post-natal and community support interventions in the strategic plan, despite the existing frameworks elsewhere [9, 10].

An effective continuum of care is expected to have a positive impact on maternal (and child) health outcomes [2, 3, 11]. It is important, therefore, to use the established frameworks to measure and monitor progress in improving women’s access to the continuum care. Progress in implementing a continuum of care can be measured and monitored at the district level, as this is the “heart” of efforts to improve maternal and new-born services in South Africa and other LMICs [12]. The district encompasses the family/community, outpatient primary centres and maternity units, and the district hospital on the continuum of care for maternal health (Fig. 2). It is the focus of primary health care re-engineering efforts in South Africa, which aim to increase access to good quality care at the district level [2, 11, 13]. However, there is no defined measurement approach to assess how well districts are performing in providing an effective continuum of care.

Researchers and policymakers use a variety of ways to measure and monitor access to the continuum of care for maternal and child health. These are in the form of various indicators that allow public health decision makers to track performance over time and across geographical areas. There is a lack of research for continuum of care measurement approaches in the South African context. This study was a systematic review of literature to explore the maternal continuum of care measurement strategies in public health research and practice among LMICs. The aim was to characterize how maternal continuum of care access was conceptualized and measured; and to describe the implications for a measurement approach in the South African health system. This measurement approach will guide and contribute to monitoring efforts and future research related to the performance of the district health system and evaluation of maternal health outcomes.

## Methods

### Study design

We conducted a critical interpretive synthesis to systematically select studies, synthesize the findings and critically assess the evidence [14]. Critical interpretive synthesis (CIS) is a review of both qualitative and quantitative studies, and the outcome is a qualitative synthesis of the evidence. The main outcomes of a critical interpretive synthesis are new *constructs* based on the evidence, and used to further the understanding of phenomena under study. CIS also uses an iterative, dynamic process to refine the review question and further develop the emerging constructs. We thus used a systematic process to first review the evidence and formulate constructs, and an additional literature review to explore their meaning and application in a specific context. The study selection, review and evidence synthesis was conducted in the period November 2016 – June 2017.

### Sampling and study selection

We conducted a search of peer reviewed articles, published or translated to English, on Academic Search Complete (EBSCO), MEDLINE (Pubmed), Cambridge Journals Online, Credo Reference and Science Direct. The initial review question was: *What indicators, methods, conceptual models and theories are used in the measurement of maternal health service delivery (including quality) and continuum of care in LMICs including South Africa?* The following key words were used as prompts: *continuum of care measurement, continuum of care model, continuum of care index, composite health measure, health service access measurement, adequacy of care measure, quality of health care measures, maternal health care quality, maternal health care coverage, maternal health care measure, care coverage measure, coverage of care models, and quality of care models*. Additional studies were also searched from the bibliographies of the studies selected by the key words [15]*.* We searched more literature during the analysis phase to validate the emerging constructs.

**Fig. 3:**
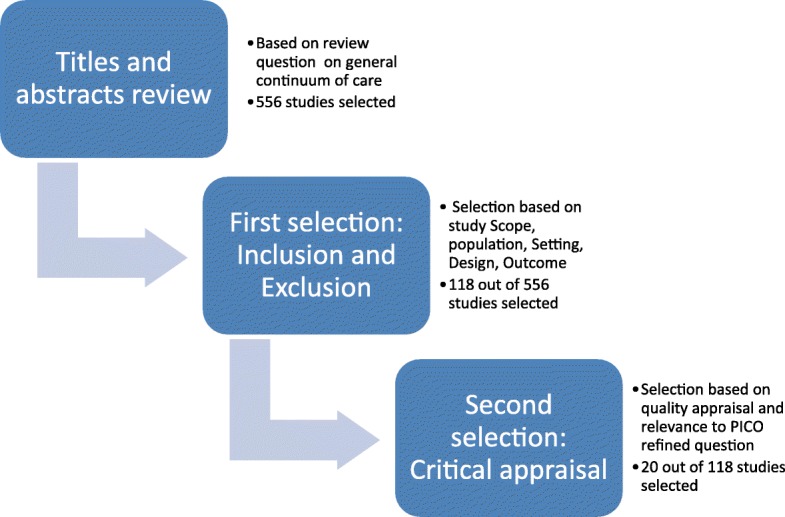
The process of selecting literature into the review

The process of systematic selection of literature is presented in Fig. 3. There were 556 articles selected after a search and review of titles and abstracts from the literature databases. We then assessed these articles according to the inclusion and exclusion criteria outlined in Table 1.

**Table 1:** Main inclusion and exclusion criteria for studies selected in the review

Main Criteria	Specific Criteria
Inclusion:	
*Scope*	Focus on: i) maternal continuum of care, or ii) adequacy of maternal healthcare service, or iii) the incorporation of quality into maternal continuum of care implementation or measurement, or iv) quality of care for maternal health within a continuum of care context
*Population*	Studies are focused on women of reproductive age Studies can also include perinatal population (maternal and neonatal health if they are relevant in scope
*Setting*	Observational studies: Low and middle income countries Theoretical studies: relevance to scope Setting may include community care if discussed in terms of continuum of care or integration into the health system Studies may be based in health systems, both health system and community care context, community care or population care (where relevant in scope)
*Outcome Variables*	The study is focused on continuum of care in maternal health, and other conceptual and theoretical approaches in reproductive health and maternal, neonatal, and child health is relevant. Non-maternal health indicators and models of continuum of care are outside the scope of this study
*Time Period*	No publication date limit
*Language*	Articles originally written in English, or an English translation from the original language if available
Exclusion:	
*Study design*	Opinion pieces/commentaries/letters to editors within inclusion scope
*Scope*	General maternal health service access and utilization literature outside of inclusion scope

Criteria guidelines based on [56]

The criteria included assessment of scope, population, outcomes of interest, and healthcare and country settings. We retained 118 articles after applying the inclusion and exclusion criteria. We then conducted a second relevance appraisal using a refined review question, more specific to continuum of care measurement and excluding other measures of health service delivery. This kind of iteration is characteristic of critical interpretive synthesis [14]. We used PICO (Population, Intervention, Comparator and Outcome) guidelines to re-define the review question [16]: “*How is the continuum of care (including quality thereof) to improve maternal health measured, for women of child-bearing age, in LMICs?”* Using the PICO guided question, a total of 20 studies were selected for quality appraisal and data extraction.

### Quality appraisal

We appraised the 20 selected studies in terms of methodological rigour, using a checklist that was based on both qualitative and quantitative research guidelines [17–20]. The studies were assessed on general areas of findings, design and sampling, analysis, reporting and interpretation, and ethics. In addition, we assessed qualitative studies on reflexivity and neutrality, and quantitative studies on risk of bias (Additional file 1). Qualitative and quantitative studies were scored out of 12 points each, and mixed methods studies out of 13. The 20 studies assessed scored between 7 and 11 out of 12 for traditional designs, and 10–11 out of 13 for the mixed methods: Scoring over 50%, all studies were thus included in the data analysis and synthesis.

### Data extraction and synthesis

There were 4 qualitative, 4 mixed methods and 12 quantitative studies included in the review. We extracted data onto a form that summarized the methods and design, key themes/indicators/models related to continuum of care measurement, interpretations and recommendations by authors, and our interpretations as investigators [21, 22]. This form is attached as Additional file 2. The forms were then uploaded to ATLAS.ti 8.0, a qualitative analysis software, for data coding and synthesis [23]. The critical interpretive synthesis relies on techniques similar to meta-ethnography in qualitative studies, in this case applied to mixed method evidence [14]. The evidence from the 20 articles is aggregated in Additional file 3 and includes methodology, metrics and indicators, and general findings. We focus our findings on the interpretation of this evidence and describe the main constructs of coverage, effective coverage and quality. We then formulated a new, synthetic construct *of adequacy of maternal continuum of care* as a result of evidence synthesis and critical reflection. *Synthetic constructs* provide the higher level explanation or clarity of a phenomenon that doesn’t currently exist in the evidence [14]. We used the synthetic and other constructs to make an argument about the evidence implications to the South African context.

## Results

The basis of our synthesis is the evidence summarized in Additional file 3, and it informs our interpretations of the metrics and methods used to measure the continuum of care for maternal health. Since the continuum of care framework was developed [3], researchers have described country specific frameworks and defined indicators for coverage and quality. The number of coverage indicators differs across studies, but consistently includes the “triad” - antenatal care visits, skilled birth attendance and post natal care check-ups [24–31]. Other indicators are related to reproductive health, newborn and child health, as well as the intersectoral factors/social determinants of health along the continuum [25–27, 29, 32–34]. Researchers have also developed varied indicators and indices to monitor quality of care along the continuum [24, 35–38]. These indicators focus mainly on content of care or performance of signal functions across continuum interventions. Lavender et al. (2016) provides a general review of quality frameworks and suggests that other aspects of quality are also important; these include women’s experiences of care and organizational factors such as staffing and resources [38]. The non-medical indicators of quality along the continuum were explored in only 2 studies in our review [35, 36]. Studies relied on different data sources to measure coverage and quality along the continuum, including Demographic and Health Surveys, routine health information systems and other household and facility data (Additional file 3). Many studies in this review used mathematical and statistical modelling methods to develop metrics related to the continuum of care, estimate trends and assess relationships [28, 29, 34, 35, 37, 39].

### Composite coverage metrics

Composite coverage metrics are used to monitor women’s access to available maternal health services on the continuum [34, 39]. Coverage estimates are often measured per intervention, while composite metrics reflect a combination of indicators across different continuum packages. The composite coverage metrics found in this review include the Composite Coverage Index [39], Co-Coverage Index [39], and the Coverage Gap Index [34]. Each index consists of a set of indicators representing interventions along the continuum. While possessing different sets of indicators, the indices are nonetheless applied to similar monitoring goals for public health research and practice. For example, Wehrmeister et al. (2016) found the Co-Coverage Index and the Composite Coverage Index to be correlated with each other and used to monitor Reproductive, Maternal, Neonatal and Child Health in similar health care settings [39]. Although they include some maternal health indicators, both of these indices were associated only with neonatal and child health outcomes [39]. The Coverage Gap Index also included many indicators of child health related to immunizations and treatment of childhood illnesses [34].

### Composite quality metrics

In order to measure quality of care along the continuum, researchers sometimes used *effective coverage* as a metric [24, 35, 40]. Effective coverage measures the coverage of interventions delivered with high quality. It is similar to the idea of “quality contacts”, which are often lower in coverage than overall contact/ visitations [24]. Effective coverage can be assessed with not just performance of signal medical functions but can include “non-medical” aspects of quality as well [35]. Effective coverage can be measured for each stage of the maternal lifecycle [24] or for whole facilities [35]. The concept is thus applied differently by researchers, using different indicators in different study settings.

The Quality Index is similar to effective coverage measurement in that it is concerned with indicators of content of care, all aggregated into a single metric [37]. The index was developed using demographic and health survey indicators across antenatal, perinatal and post-natal care. The non-medical aspects of quality included in the metric were related to health provider-client communication about delivery preparedness. The authors recommended inclusion of more indicators of care and communication across the wider continuum of care.

### The adequacy of continuum of care for maternal health

Our review revealed gaps in the measurement of the continuum of care for maternal health in terms of i) composite metrics that include both coverage and quality, ii) composite metrics that include more maternal health components, and iii) multi-dimensional quality measurement. In order to address these gaps, we used the evidence in the review to explore a more holistic measure of the continuum of care for maternal health, termed *adequacy*. Adequacy will address more maternal health indicators, the integration of quality measures, and the intersectoral factors across the continuum.

As an iterative process, CIS allowed us more qualitative review of literature to explore the meaning of the emerging construct of adequacy [14, 41]. Adequacy has been applied to evaluation of antenatal care programs [42–45]; assessment of human and health system resources [6, 46] and overall performance [47]; assessment of dimensions of care [12]; and evaluation of impact of interventions [48, 49]. Adequacy is often expressed as a count or combination of interventions that produce positive effects on maternal health and related outcomes. From our continuum of care perspective therefore, adequacy would reflect the *collective* threshold of interventions needed to produce positive maternal health outcomes. This collective can be measured via metrics that comprise indicators across packages of care and calculated by statistical methods.

Other authors have measured adequacy within a single package of care and investigated how it affects utilization of services along the continuum [29]. In that study, adequacy was a latent construct consisting of indicators of effective coverage similar to those discussed earlier. Our concept of adequate continuum of care for maternal health focuses on the collective performance of indicators across packages of care. The Kerber framework and its adaptations are useful for guiding the types of interventions to be provided along the continuum of care for maternal health [3, 7]; whereas an adequacy framework can help lay out a measurement approach for monitoring integrated service provision at district levels. Although indicators of adequacy will depend on a thorough assessment of data sources at district levels, the evidence in this review can provide us with a tentative definition of the approach as:

*Measurement of timely access to evidence based interventions encompassed by the continuum of care service provision framework for maternal and child health, through a positive experience of care and within a supportive structural context that ensures good quality of care (*i.e. *competent human resources, actionable information systems, functional referral systems and essential physical resources).*

Adequacy is thus a patient-centred measure, even though in practice many of the indicators are measured from the supply side.

## Discussion

This critical interpretive synthesis revealed that *coverage*, *effective coverage* and *quality* were the main measurements made on the continuum of care for maternal health. Coverage is a measure of access to healthcare among those who need it, as opposed to mere availability of services [3, 50]. It can be calculated for specific interventions or packages along the continuum of care [26, 27, 30, 33]. Composite metrics or indices of coverage cover a combination of interventions across packages [34, 39]. The composite indices found in this study were reflective of newborn and child health outcomes, while overall indicators varied across studies. This makes them unsuitable for measurement of coverage along the continuum of care for maternal health.

The studies in this review measured quality through a detailed assessment of sets of often numerous indicators [36, 40], or through composite metrics such as effective coverage and the Quality Index [35, 37]. These quality metrics focused on content of care and, to a lesser extent, its “context”. Context of quality of care is defined by organizational factors and patient experience of care [38]. There are frameworks such as the WHO Quality of Care Framework for Maternal and Newborn Health [38, 51] which capture these factors in terms of physical and human resources, referral and information systems, and physical infrastructure. Our definition of an adequate continuum of care for maternal health incorporates the measurement of coverage and the multidimensional quality of care to influence health outcomes.

Besides coverage and quality, an integrated measure of continuum of care adequacy will include the social determinants of health largely missing from existing measures. The concept of timeliness is also crucial to the definition of adequacy of care delivered [29, 42]. The adequacy of continuum of care for maternal health can be measured at the health district level in South Africa, as it encompasses all of the framework interventions needed to improve maternal health outcomes [2, 7, 13]. Horizontal metrics of adequacy at each level of care can help measure performance at that level and support localized decision making. Diagonal metrics that include all levels from family/community to the district level can reflect overall health district performance. These applications of adequacy would be consistent with how current metrics are measured and applied, and will shed more light on maternal health at subnational level.

Studies in this review depended on various data sources to identify or derive indicators for coverage and/or quality along the maternal continuum of care. These data sources included household surveys, community health surveillance, and routine health information system data. In the South African context, adequacy can be measured through triangulation of all these different data sources. Data source interpolation has been recommended as one strategy to improve data sufficiency: for instance, data from population surveys can be supplemented with routine health information system to provide more accurate estimations of coverage and include a wider range of indicators [24, 50]. Examples of these data sources in South Africa are the District Health Information System [52, 53]; Demographic and Health Survey and intersectoral factor data from the national statistics body, Statistics South Africa [54]. Health and socio-demographic surveillance systems such as Agincourt Health and Demographic Surveillance System are also vital sources of data on the life course, which help evaluate coverage and impact of both health and social interventions [55]. These are theoretical applications however, as a detailed assessment of South African health information systems is needed to derive indicators and model the adequacy metrics. The focus of the review was the conceptual backgrounds, indicators and metrics used to measure the continuum of care, and thus we don’t delve into broader aspects of monitoring related to policy and implementation.

## Conclusion

Current measurement approaches for the maternal health continuum of care are neither integrated enough nor immediately applicable to the South African context. Their applicability is limited by variability among the metrics, lack of comprehensiveness and the association with child rather than maternal health outcomes among others. Critical interpretive synthesis helped us synthesize the evidence on these approaches and propose a useful way forward. We conceptualized adequacy as an integrated measure of quality, coverage and the social determinants of health/intersectoral factors. This integrated approach will address some of the challenges observed in the current measures, and is focused on maternal health. We discussed the potential configuration of adequacy metrics based on the findings of the synthesis. However, more research is needed to derive indicators from the proposed data sources and formulate metrics using the appropriate methods.

## Additional files

Additional file 1: The quality assessment criteria for studies selected into the review. The scoring guide for each study in the review based on essential criteria and key indicators/questions for each study design. (DOCX 18 kb)

Additional file 2: Tool used to extract data from the articles selected in the critical interpretive synthesis. Includes aims of the study, theoretical framework, data analysis approach, key indicators, and main findings among others. (DOCX 19 kb)

Additional file 3: Summary of main findings, methodology and metrics/indicators identified among all the articles reviewed. Summary for the 20 articles selected into the review. (DOCX 50 kb)

## Abbreviations

ANC: Ante Natal Care
CIS: Critical Interpretive Synthesis
COC: Continuum of Care
DHIS: District Health Information System
DHS: Demographic and Health Survey
EmOC: Emergency Obstetric Care
IMPAC: Integrated Management of Pregnancy and Childbirth
LMIC: Low and Middle Income Country
LQAS: Lot Quality Assurance Sampling
MCH: Maternal and Child Health
MNCH: Maternal Newborn and Child Health
PMNCH: Partnership for Maternal, Newborn and Child Health
PNC: Post Natal Care
RMNCH: Reproductive, Maternal, Neonatal, and Child Health
SBA: Skilled Birth Attendance
WHO: World Health Organization
