# Supplementary material for: Measurement approaches in continuum of care for maternal health: a critical interpretive synthesis of evidence from LMICs and its implications for the South African context
Source: BMC Health Serv Res. 2018 Jul 11;18:539. doi: 10.1186/s12913-018-3278-4 (PMC6042348; doi:10.1186/s12913-018-3278-4)
Supplement: Supplementary file 1 — The quality assessment criteria for studies selected into the review. The scoring guide for each study in the review based on essential criteria and key indicators/questions for each study design. (DOCX 18 kb) [file 12913_2018_3278_MOESM1_ESM.docx]

Additional file 1 The quality assessment criteria for studies selected into the review

| **Study Design** | **Quality Area** | **Essential Criteria** | **Key Indicators/Questions** | **Scoring** |
| --- | --- | --- | --- | --- |
| Qualitative and Quantitative | *Findings/Results* | - Credibility - Expansion of knowledge - Generalizability/inferences explained | - Findings/conclusions ‘make sense’/have a coherent logic - Findings presented or conceptualized in a way that offers new insights/alternative ways of thinking - Discussion of limitations of evidence - Discussion of what can be generalised to wider population OR description of the contexts in which the study was conducted to allow applicability to other settings/contextual generalities to be assessed | 3 |
|  | *Design and Sampling* | - Method/design consistent with research intent - Sample and sampling method appropriate - Data collection strategy apparent and appropriate | - Rationale explored for specific method - Use of different features of design evident in findings presented - Discussion of limitations of design and their implications for the study evidence - Description of how sampling was undertaken - Justification for the sampling strategy provided - Description of how data was collected - Methods appropriate for type of data required. | 3 |
|  | *Analysis* | - Approach to analysis conveyed well - Analytical approach appropriate | - Clear rationale for choice of data management method/tool/package - Analytical approach appropriate for the method chosen - Discussion of how coding systems/conceptual frameworks evolved (qualitative) | 2 |
|  | *Reporting/Interpretation* | - How clear and coherent is the reporting? - Link between data, interpretation and conclusions clear | - Key messages highlighted or summarized - Discussion of how explanations/ theories/conclusions were derived | 2 |
|  | *Ethics* | - Demonstration of sensitivity to ethical concerns | - Informed Consent - Confidentiality of data, respect/protection of participants including anonymity - Ethical committee approval | 1 |
| Qualitative (additional) | *Reflexivity & Neutrality* | - Assumptions that shape the output of the evaluation clear | - Discussion/evidence of the main assumptions/hypotheses/theoretical ideas on which the evaluation was based - Evidence of openness to new/alternative ways of viewing subject/theories/ assumptions - Discussion of how error or bias may have arisen in design/data collection/analysis and how addressed, if at all - Reflections on the impact of the researcher on the research process | 1 |
| Quantitative (additional) | *Risk of Bias* | - Measures to reduce bias in RCT   OR   - Measures to reduce bias in observational studies | - Allocation concealment - Blinding - Complete accounting of participants and outcomes - Non-selective outcome reporting - Use of validated outcome measures   OR   - Appropriateness of eligibility criteria - Sound/non-differential measurement of exposure and outcome among populations of interest - Description, measurement and control of confounding - Adequate follow up of both cases and control - Discussion of how error or bias may have arisen in design/data collection/analysis and how addressed, if at all | 1 |
|  |  |  |  |  |
|  |  |  | **Total Maximum Points (Qualitative)** | 12 |
|  |  |  | **Total Maximum Points (Quantitative)** | 12 |
|  |  |  | **Total Maximum Points (Mixed)** | 13 |
