# Supplementary material for: Measurement approaches in continuum of care for maternal health: a critical interpretive synthesis of evidence from LMICs and its implications for the South African context
Source: BMC Health Serv Res. 2018 Jul 11;18:539. doi: 10.1186/s12913-018-3278-4 (PMC6042348; doi:10.1186/s12913-018-3278-4)
Supplement: Supplementary file 2 — Tool used to extract data from the articles selected in the critical interpretive synthesis. Includes aims of the study, theoretical framework, data analysis approach, key indicators, and main findings among others. (DOCX 19 kb) [file 12913_2018_3278_MOESM2_ESM.docx]

Additional file 2 Tool used to extract data from the articles selected in the critical interpretive synthesis

| *Citation:* |  |
| --- | --- |
| *Country:* |  |
| *Aims of the Study* |  |
| *Ethics – how ethical issues were addressed:* |  |
| *Study setting:* |  |
| *Theoretical background of study:* |  |
| *Sampling approach:* |  |
| *Participant characteristics:* |  |
| *Data collection methods:* |  |
| *Data analysis approach:* |  |
| *Number and types continuum indicators:* |  |
| *Key themes/indicators/models identified in the study* |  |
| *Findings/Data extracts related to the key themes:* |  |
| *Author explanations of the key themes/indicators/models* |  |
| *Methodological and Conceptual recommendations made by authors:* |  |
| *Reviewer Interpretation* |  |
| *Assessment of study quality (rating from Table 1):include score and specific remarks on study quality* |  |

**Based on**

**1.** Munro S, Lewin S, Smith H, Engel M, Fretheim A, Volmink J. (2007) *Adherence to tuberculosis treatment: a qualitative systematic review of stakeholder perceptions*. PLOS Medicine. 4(7): e238

2. Noyes J & Lewin S. Chapter 5: Extracting qualitative evidence. In: Noyes J, Booth A, Hannes K, Harden A, Harris J, Lewin S, Lockwood C (editors), *Supplementary Guidance for Inclusion of Qualitative Research in Cochrane Systematic Reviews of Interventions*. Version 1 (updated August 2011). Cochrane Collaboration Qualitative Methods Group, 2011. Available from URL <http://cqrmg.cochrane.org/supplemental-handbook-guidance>
