# Supplementary material for: Measurement approaches in continuum of care for maternal health: a critical interpretive synthesis of evidence from LMICs and its implications for the South African context
Source: BMC Health Serv Res. 2018 Jul 11;18:539. doi: 10.1186/s12913-018-3278-4 (PMC6042348; doi:10.1186/s12913-018-3278-4)
Supplement: Supplementary file 3 — Summary of main findings, methodology and metrics/indicators identified among all the articles reviewed. Summary for the 20 articles selected into the review. (DOCX 50 kb) [file 12913_2018_3278_MOESM3_ESM.docx]

Additional file 3 Summary of main findings, methodology and metrics/indicators identified among all the articles reviewed

| **Citation** | **Main Findings** | **Methods** | **Continuum of Care Metrics/Indicators** |
| --- | --- | --- | --- |
| [1] | Measurement of coverage along the continuum of care (COC) for maternal, newborn and child health (MNCH) should be conducted using complementary data sources, and should include measurement of quality. | Qualitative review of literature | Studies reviewed focused on different indicators around Antenatal Care (ANC), birth interventions, vaccination, treatment of childhood illnesses |
| [2] | A customized COC model for MNCH in Nepal is feasible | Review of literature and consultations with experts in the country. | Contraceptive Prevalence Rate (CPR), ANC, Tetanus for pregnant woman, Delivery at health institution, Skilled birth attendance (SBA), Exclusive breastfeeding, Measles immunization, Antibiotics for suspected pneumonia, Oral Rehydration Therapy (ORT) for under 5 years old diarrhoea case |
| [3] | A population level framework can be defined that outlines integrated service delivery packages along the COC for MNCH | Qualitative review of literature | No indicators but a framework of general packages of interventions including family/community care, reproductive health, ANC childbirth care, PNC, newborn and child care, and child health. |
| [4] | Two different indices for coverage along the MNCH continuum are correlated with each other and some child and neonatal outcomes. | Quantitative analysis of coverage and correlations; stratifications by wealth quintiles. | Co-coverage Index (8 preventive interventions for mother and child: ANC, tetanus during pregnancy, SBA, vaccinations and supplements for child, access to improved drinking water)  Composite Coverage Index: family planning, SBA, vaccinations for child, ORT for diarrhea, and care seeking for pneumonia. |
| [5] | There were district gaps in MNCH coverage measured using the Coverage Gap Index (CGI). Areas with high gaps (>=50%) identified. | Use of household and facility survey data to calculate the (CGI) in Indian districts | CPR, SBA, ANC, Immunizations (measles; Diphtheria, pertussis and tetanus; BCG), ORT, Treatment for Acute Respiratory Infections (ARI) |
| [6] | Quality in emergency obstetric (EmOC) and newborn care (EmNC) was low in a sample of health facilities in Ghana. Facilities performed slightly better on routine signal functions. | Health facility assessment; quantitative analysis of effective coverage and composite quality measures. | Many signal functions of Routine Delivery Care, EmOC and EmNC basic and comprehensive functions; as well as non-medical aspects of quality (mainly hygienic toilet conditions) |
| [7] | 193 indicators developed for routine information systems to monitor WHO essential interventions for RMNCH. Household surveys not sufficient. | Indicators derived from surveys and routine information systems, and evaluated by panel of experts. | Framework of 193 indicators across 45 WHO Essential Interventions from preconception to newborn care. |
| C. [8] | Differences in quality EmOC and EmNC performance among hospitals and health facilities in three African countries. High access and low quality of care concomitant. | Facility quality and coverage assessment tools; calculation of indicators. | Availability of EmOC and EmNC facilities, fatality rates, and performance of Caesareans; Equity indicators; Multi-dimensional quality indicators. |
| [9] | Creation of a quality index from demographic and health survey (DHS) data is feasible. The index is associated with disparities in quality of care by wealth and geographical location. | Used WHO guidelines to identify indicators. Created composite indices using statistical methods. Explored association with equity markers. | ANC visits, signal functions during ANC, supplementation and immunizations during pregnancy, discussion about birth preparedness and recognition of danger signs, signal functions for newborn and postnatal checks. |
| [10] | There are many frameworks for assessing quality of care and provider competency, organizational factors and women’s perspectives should all be considered in monitoring. | Narrative review of current quality frameworks | Core essential interventions for childbirth and postnatal care (PNC) for mother and newborn, women’s experience of care, provider competency. |
| [11] | When the content of care is measured, coverage of high quality contacts was consistently lower than crude coverage among facilities in three low and middle income countries (LMICs). | Households and frontline health worker surveys to estimate coverage by specific interventions | 8 criteria of routine processes performed during ANC visit; 2 criteria for prevention of haemorrhage during SBA; 5 criteria for post-partum care; 5 criteria for PNC. |
| [12] | Moderate improvements in COC coverage indicators in a program intervention area in Nigeria. | Random sample of households in intervention area interviewed at baseline and follow up. | Demand for family planning (FP), ANC, SBA, PNC for mother and baby, exclusive breastfeeding, vaccination for DPT3, and antibiotics for pneumonia. |
| [13] | Testing a model of the MNCH COC in community settings in Ghana. | A cluster randomized controlled trial design to measure effectiveness and implementation outcomes | COC completion, PNC care delivered, complications, perinatal and neonatal mortality, coverage of interventions, costs and sustainability. |
| [14] | Coverage targets were low for MNCH COC interventions nationally and sub-nationally in Nigeria. Intervention improved coverage some. | LQAS methodology to sample study areas. Composite coverage rates estimated per study area. | Demand for FP, ANC, SBA, PNC, exclusive breastfeeding, DPT3, and antibiotics for pneumonia . |
| [15] | Coverage of interventions along the MCH continuum differed across subnational levels, with urban/rural disparities also observed. | Estimated indicators by statistical models using household, administrative and census data. | Health outcomes; MCH interventions – ANC, tetanus, SBA, Breastfeeding, ORS, care for ARI,; Socioeconomic factors – housing, electricity, sanitation, water, education |
| [16] | Use of one COC level influences progression to another. Socioeconomic indicators also influence use of the COC services. | Descriptive and regression analysis methods on DHS data. | ANC one or four visits, SBA, PNC as well as family planning counseling within year of birth. |
| [17] | A majority of women in Nigeria dropped out of the COC from ANC to PNC due to socio-economic issues and lack of access to facilities. | Descriptive and regression analysis methods on DHS data. | ANC, SBA, PNC. |
| [18] | Positive associations from one adequate level of care to another. Low utilization of some levels of the MNCH COC in a number of Sub-Saharan Africa countries. | Structural equation modeling of relationships in the COC. | Child immunization, PNC for mother and baby, delivery care (including caesarean services and SBA), ANC (including specific signal functions). Various individual and community factors as determinants of utilizat ion. |
| [19] | Regional variations in utilization of different stages of the MCH COC in Cambodia | Descriptive statistics. Regression models to assess factors that affect use of COC services. | ANC, SBA and PNC and their independent determinants. |
| [20] | COC completion rates were low in Ghana, with various factors affecting utilization. | Descriptive statistics and multiple logistic regression models. | ANC, SBA and PNC and their independent determinants |

[1] J. Bryce, F. Arnold, A. Blanc, A. Hancioglu, H. Newby, and J. Requejo, “Measuring Coverage in MNCH : New Findings , New Strategies , and Recommendations for Action,” *PLoS Med.*, vol. 10, no. 5, 2013.

[2] K. Ashish *et al.*, “State of maternal , newborn and child health programmes in Nepal : what may a continuum of care model mean for more effective and efficient service delivery?,” *J. Nepal Heal. Res. Counc.*, no. March 2015, 2011.

[3] K. J. Kerber, J. E. de Graft-Johnson, Z. A. Bhutta, P. Okong, A. Starrs, and J. E. Lawn, “Continuum of care for maternal, newborn, and child health: from slogan to service delivery,” *Lancet*, vol. 370, no. 9595, pp. 1358–1369, 2007.

[4] F. C. Wehrmeister, M. Restrepo-mendez, V. A. Franca, and G. Victora, “Summary indices for monitoring universal coverage in maternal and child health care,” *Bull. World Health Organ.*, vol. 94, no. November, pp. 903–912, 2016.

[5] R. K. Rai, C. Kumar, and P. K. Singh, “District level coverage gap in Maternal, Newborn and Child Health care services in India,” *J. Epidemiol. Glob. Health*, vol. 2, no. 4, pp. 221–224, 2012.

[6] R. C. Nesbitt *et al.*, “Quality along the Continuum : A Health Facility Assessment of Intrapartum and Postnatal Care in Ghana,” *PLoS One*, vol. 8, no. 11, pp. 1–11, 2013.

[7] V. Flenady *et al.*, “eRegistries : indicators for the WHO Essential Interventions for reproductive , maternal , newborn and child health,” *BMC Pregnancy Childbirth*, vol. 16, no. 293, pp. 1–15, 2016.

[8] C. Wilunda, G. Putoto, D. D. Riva, and F. Manenti, “Assessing Coverage , Equity and Quality Gaps in Maternal and Neonatal Care in Sub- Saharan Africa : An Integrated Approach,” *PLoS One*, vol. 10, no. 5, pp. 1–16, 2015.

[9] Z. Dettrick, H. N. Gouda, A. Hodge, and E. Jimenez-Soto, “Measuring quality of maternal and newborn care in developing countries using demographic and health surveys,” *PLoS One*, vol. 11, no. 6, pp. 1–21, 2016.

[10] D. T. Lavender, “Improving quality of care during labour and childbirth and in the immediate postnatal period,” *Best Pract. Res. Clin. Obstet. Gynaecol.*, vol. 36, pp. 57–67, 2016.

[11] T. Marchant *et al.*, “Adding content to contacts: Measurement of high quality contacts for maternal and newborn health in Ethiopia, North East Nigeria, and Uttar Pradesh, India,” *PLoS One*, vol. 10, no. 5, pp. 1–20, 2015.

[12] D. Abegunde, N. Orobaton, H. Sadauki, and A. Bassi, “Countdown to 2015 : Tracking Maternal and Child Health Intervention Targets Using Lot Quality Assurance Sampling in Bauchi State,” pp. 1–14, 2015.

[13] K. Kikuchi *et al.*, “Ghana’s Ensure Mothers and Babies Regular Access to Care (EMBRACE) program: study protocol for a cluster randomized controlled trial.,” *Trials*, vol. 16, no. 1, p. 22, 2015.

[14] D. Abegunde *et al.*, “Monitoring maternal, newborn, and child health interventions using lot quality assurance sampling in Sokoto State of northern Nigeria,” vol. 9716, no. April 2017, 2015.

[15] D. A. Roberts *et al.*, “Benchmarking health system performance across regions in Uganda : a systematic analysis of levels and trends in key maternal and child health interventions , 1990 – 2011,” *BMC Med.*, vol. 13, no. 285, pp. 1–17, 2015.

[16] K. Singh, W. T. Story, and A. C. Moran, “Assessing the Continuum of Care Pathway for Maternal Health in South Asia and Sub-Saharan Africa.,” *Matern. Child Health J.*, vol. 20, no. 2, pp. 281–9, Feb. 2016.

[17] J. O. Akinyemi, R. F. Afolabi, and O. A. Awolude, “Patterns and determinants of dropout from maternity care continuum in Nigeria.,” *BMC Pregnancy Childbirth*, vol. 16, no. 1, p. 282, 2016.

[18] P. O. Owili, M. Adoyo Muga, Y.-J. Chou, Y.-H. Elsa Hsu, N. Huang, and L.-Y. Chien, “Associations in the continuum of care for maternal, newborn and child health: a population-based study of 12 sub-Saharan Africa countries,” *BMC Public Health*, vol. 16, pp. 1–15, 2016.

[19] W. Wang and R. Hong, “Levels and determinants of continuum of care for maternal and newborn health in Cambodia- evidence from a population-based survey,” *BMC Health Serv. Res.*, vol. 15, no. 62, pp. 1–9, 2015.

[20] F. Yeji *et al.*, “Continuum of Care in a Maternal, Newborn and Child Health Program in Ghana: Low Completion Rate and Multiple Obstacle Factors,” *PLoS One*, vol. 10, no. 12, pp. 1–24, 2015.
